# Lack of Cholesterol Awareness among Physicians Who Smoke

**DOI:** 10.3390/ijerph6020635

**Published:** 2009-02-11

**Authors:** Richard E. Scranton, Wildon R. Farwell, John M. Gaziano

**Affiliations:** 1 Division of Aging, Department of Medicine, Brigham and Women’s Hospital, Harvard Medical School, 1334 Main Road, Tiverton, RI 02878, Boston, MA, USA; 2 Massachusetts Veterans Epidemiology Research and Information Center, VA Boston Healthcare System, Boston, MA, USA; 3 Division of Preventive Medicine, Department of Medicine, Brigham and Women’s Hospital, Harvard Medical School, Boston, MA, USA

**Keywords:** Disease prevention, cholesterol, lifestyle behavior, tobacco smoking

## Abstract

Cigarette use is a known risk factor for the development of coronary artery disease (CAD) as it adversely affects HDL cholesterol levels and promotes thrombogenesis. Smoking may also be associated with behavioral characteristics that potentiate the risk of CAD. A lack of cholesterol knowledge would indicate an aversion to a prevention-oriented lifestyle. Thus, our goal was to determine the association between tobacco use and knowledge of self-reported cholesterol among male physicians. Using the 1982 and follow-up questionnaires from the physician health study, we report the changes in the frequencies of awareness of self-reported total cholesterol and cardiovascular risk factors among the 22,067 participants. We classified physicians as being aware of their cholesterol if they reported a cholesterol level and unaware if the question was left unanswered. In 1997, 207 physicians were excluded, as the recorded cholesterol was not interpretable, leaving 21,860 for our follow up analyses. Using unadjusted logistic models, we determined the odds ratios (OR) and 95% confidence intervals (CI) of not reporting a cholesterol level in either 1982 or 1997 for each specified risk factor. We then evaluated whether the lack of cholesterol awareness at both time points was associated with the use of tobacco throughout the study. After 14-years of follow up, cholesterol awareness increased from 35.9 to 58.6 percent. During this period, the frequency of hypertension and hyperlipidemia treatment increased (13.5 to 40.5% and 0.57% to 19.6% respectively), as did the diagnosis of diabetes (2.40 to 7.79%). Behavioral characteristics such as a sedentary lifestyle and obesity also increased (27.8 to 42% and 43.5 to 53.5%, respectively), however the proportion of current smokers deceased from 11.1 to 4.05%. The percentages of individuals being unaware of their cholesterol decreased in all risk factor groups. However, individuals were likely to be unaware of their cholesterol at both time points if they were current smokers (1982 OR 1.44, CI 1.4–1.7; 1997 OR 1.71, CI 1.48–1.97), past smokers (1982 OR 1.12, CI 1.05–1.18; 1997 OR 1.13, CI 1.06–1.20), overweight (BMI 25 kg/m2) or sedentary. In addition, physicians who never quit smoking were likely to be unaware of their cholesterol throughout the study (OR 1.42, CI 1.21–1.67). Cholesterol awareness in general and among those with CAD risk factors improved after 14-years of follow-up. However, the likelihood of being unaware was greater among smokers at both time points. Therefore, smokers do not appear to take advantage of other preventive strategies that would minimize their risk of developing CAD.

## 1. Introduction

Cardiovascular disease is a significant cause of morbidity and mortality in the United States. Smoking increases the risk for cardiovascular disease by decreasing levels of high-density lipoprotein cholesterol and promoting thrombogenesis [1,2]. In addition, patients who smoke may possess psychological traits such as hostility more frequently than non-smokers [3]. These traits have also been found to increase the risk of cardiovascular disease.

Because smokers are at higher risk for cardiovascular disease, smokers could potentially benefit from adoption of a prevention-oriented lifestyle. Characteristics of a prevention-oriented lifestyle would include identifying risk factors for chronic medical disease such as cardiovascular disease and adopting risk reduction strategies [4]. Cholesterol is a known risk factor for cardiovascular disease and national guidelines recommend routine screening for hypercholesterolemia. Patients who are unaware of their cholesterol level are less likely to have elevated cholesterol treated and therefore less likely to reduce their cardiovascular risk. Little is known about the frequency of smokers to adopt a prevention-oriented lifestyle. Therefore, we examined the frequency of cholesterol unawareness, as a surrogate for failure to adopt a prevention-oriented lifestyle, among physicians in the Physicians Health Study.

## 2. Methods

Our study population consisted of physicians who participated in the physicians’ health study (PHS) I and II. The PHS I was a randomized trial of aspirin and beta-carotene in the primary prevention of cardiovascular disease and cancer and consisted of 22,067 male physicians aged ≥ 40 years [5]. The PHS II is a randomized trial of beta-carotene, vitamin E, vitamin C, and a multivitamin in the prevention of cardiovascular disease, cancer, and eye disease and consisted of male physicians aged ≥ 50 years [6]. In both PHS I and PHS II, physicians were asked to provide self-reported cholesterol levels on the baseline questionnaire. Since many physicians that participated in PHS I also continued in PHS II we were able to capture follow up data on most of the participants with 14 years of time between the two questionnaires. Using the baseline and follow-up (PHS 1 follow up or PHS II baseline) questionnaires, we report the changes in the frequencies of awareness of self-reported total cholesterol and cardiovascular risk factors among the 22,067 participants. Physicians were classified as being aware of their cholesterol if they reported a cholesterol level and unaware if the question was returned blank. Physicians were excluded from the analysis if their answer to the question was uninterruptable. 207 physicians were excluded from follow up, as the recorded cholesterol was not interpretable, leaving 21,860 for our follow up analyses. Additional information was collected from baseline questionnaires including self-reported age (categorized as < 65 years and ≥ 65 years), weight and height (converted to BMI, kg/m2), treatment for hypertension (yes or no), treatment for hypercholesterolemia (yes or no), history of diabetes mellitus (yes or no), smoking status (categorized as current, former, or never), frequency of physical activity (categorized as none or exercise to a sweat at least one time per week), and family history of myocardial infarction (yes or no).

We describe the frequency of cardiovascular risk factors and cholesterol awareness at baseline and follow-up. We also report the frequency of physicians who were aware of their cholesterol level among groups of cardiovascular risk factors. Unadjusted logistic regression models were used to describe the relationship between cardiovascular risk factors and awareness of cholesterol. Logistic regression models were also used to calculate the odds ratio (OR) and 95% confidence intervals (CI) of the likelihood of physicians who reported having a cardiovascular risk factor on the baseline questionnaire and who also reported being unaware of their cholesterol level at follow up compared to physicians who reported not having a cardiovascular risk factor. Logistic models were also used to determine if physicians who smoked throughout the study were also more likely to be unaware throughout the study as compared to never smokers.

## 3. Results

At the baseline 64.1% of participants were unaware of their cholesterol level whereas approximately 14 years later, 41.4% of participants were unaware of their cholesterol level (Table 1). During this period, the frequency of several cardiovascular risk factors including hypertension, hyperlipidemia, diabetes mellitus, sedentary lifestyle, and obesity increased whereas the frequency of current smokers decreased. Among physicians with cardiovascular risk factors, the frequency of cholesterol unawareness increased in every group (Table 2) as compared to physicians without cardiovascular risk factors. The frequency of cholesterol unawareness among physicians who reported being current smokers was 71.7% at the baseline and 46.2% at the follow up. This represents a drop of 35.6% in the frequency of cholesterol unawareness among physicians who reported being current smokers. However, the frequency of cholesterol unawareness among physicians who smoked was higher than in any other category of cardiovascular risk factors at the baseline and remained higher than all categories of cardiovascular risk factors except physicians with diabetes at the follow up.

Compared to physicians without a particular cardiovascular risk factor, several groups of physicians with a particular cardiovascular risk factor were more likely to be unaware of their cholesterol level (Table 3). Compared to physicians that never smoked, physicians who were current smokers were more likely to be unaware of their cholesterol at the baseline (OR = 1.55, 95% CI = 1.40 to 1.70) and follow up (OR = 1.71, 95% CI = 1.48 to 1.97). The odds of cholesterol unawareness were higher among physicians that currently smoked than among physicians in any other category of cardiovascular risk at the baseline and follow up. The odds of cholesterol unawareness were lower among physicians that were currently being treated for hyperlipidemia than among physicians in any other category of cardiovascular risk at the baseline and follow-up.

Thirty-five percent of the physicians who were unaware at baseline were now aware after 14 years of follow up and only 12% who were aware of their cholesterol at baseline were no longer aware upon follow up. Among the physicians who were always unaware (29%), they were more likely to have smoked throughout the study (OR 1.42, CI 1.21—1.67) as compared to never smokers (Table 4).

## 4. Discussion

In our study, the frequency of cholesterol unawareness among physicians who smoked was higher than among physicians with any other risk factor for cardiovascular disease at baseline and all but diabetes mellitus at follow up. The odds of cholesterol unawareness were highest among physicians who currently smoked than among physicians with any other risk factor of cardiovascular disease at baseline and follow up. The frequency of cholesterol unawareness in the overall cohort and among physicians who currently smoked declined between the baseline and follow up questionnaires.

Our study suggests that although they are at high cardiovascular risk, physicians who smoke are less likely to adopt a prevention-oriented lifestyle than are physicians with other cardiovascular risk factors. Other studies have also reported that patients who smoke are less likely to adopt a prevention-oriented lifestyle [7–11]. Although these physicians likely knew about the increased risk for cardiovascular disease by smoking, the physicians who smoked did not appear to mitigate this risk by ascertaining their risk from cholesterol as frequently as physicians with other cardiovascular risks. It is very likely that the combination of these factors and not smoking alone accounts for the fact that individuals that smoke compared to non smokers have a reduced probability for a long life [12]. It is interesting to note that physicians who were being treated for hypertension and were therefore aware of a cardiovascular risk factor were less likely to be unaware of their cholesterol than were physicians not being treated for hypertension.

Justification for the use of cholesterol unawareness as a surrogate for adoption of a prevention-oriented lifestyle is based upon previously published findings [13,14]. We previously reported that physicians who were aware of their total cholesterol level were more likely to have a lower measured total cholesterol level and ratio of total cholesterol to high-density lipoprotein cholesterol level over time.

Several limitations should be considered when interpreting the findings of our study. Although physicians reported a cholesterol level, the level that they reported may not have been accurate. For our analysis, we were only interested in whether physicians were aware of their cholesterol level. We were not interested in how accurate they were in reporting their cholesterol level. It is possible that some physicians estimated their cholesterol level without actually having had their cholesterol level measured and were therefore misclassified as being aware when in fact they were truly unaware of their cholesterol level. However, this is unlikely as physicians who are interested in having their cholesterol measured have ample resources for having it measured. Because physicians are more likely to be aware of the increased cardiovascular risk associated with smoking and have more resources to measure cholesterol, our findings may underestimate the true association between smoking and cholesterol unawareness in the general population. Finally, many changes likely occurred among the physicians after 14 years of follow up. Indeed, a large percentage of physicians that were not aware in 1982 were now aware in 1997. We did not perform sub analyses on all the potential combinations of changing awareness and changing risk factors. Since our intent was to focus on the associations between smoking and cholesterol awareness, we did evaluate changes in awareness among this subgroup. Again our findings would suggest that physicians who were current smokers during the 14-years of follow-up were also more likely to be unaware of their cholesterol throughout the study.

## 5. Conclusions

In conclusion, we found a significant positive relationship between smoking and cholesterol unawareness. Our findings suggest that smokers do not appear to adhere to a prevention-oriented lifestyle.

## Figures and Tables

**Table 1.** The frequency on the baseline and follow up questionnaire of cholesterol unawareness and cardiovascular risk factors.

Variable	Baseline	Follow-up
**Cholesterol Unawareness, n (%)**	14,154 (64.1)	9,138 (41.4)
**Age ≥ 65 years, n (%)**	3,066 (13.9)	9,245 (54.8)
**Hypertension Treatment, n (%)**	2,843 (13.5)	6,822 (40.5)
**Hyperlipidemia Treatment, n (%)**	106 (0.57)	3,264 (19.6)
**Diabetes Mellitus, n (%)**	533 (2.40)	1,719 (7.79)
**BMI ≥ 25 kg/m2, n (%)**	9,591 (43.5)	9,779 (53.5)
**Sedentary Lifestyle, n (%)**	6,056 (27.8)	8,585 (42.0)
**Family History of Myocardial Infarction, n (%)**	2,851 (13.1)	2,851 (13.1)
**Former Smoker, n (%)**	8,671 (39.4)	8,253 (42.1)
**Current Smoker, n (%)**	2,438 (11.1)	793 (4.05)

**Table 2.** Frequency of physicians unaware of their cholesterol level at the baseline and follow up among groups of cardiovascular risk factors.

Cardiovascular Risk Factor	Unaware of Cholesterol Level	
	Baseline	Follow up
**Age ≥ 65 years, %**	58.1	26.0
**Hypertension Treatment, %**	54.5	22.1
**Hyperlipidemia Treatment, %**	36.8	6.7
**Diabetes Mellitus, %**	52.9	48.3
**BMI ≥ 25 kg/m2, %**	65.2	31.9
**Sedentary Lifestyle, %**	68.0	43.9
**Family History of Myocardial Infarction, %**	62.7	36.5
**Former Smoker, %**	64.6	36.2
**Current Smoker, %**	71.7	46.2

**Table 3.** Odds of cholesterol unawareness among physicians with a cardiovascular risk factor compared to physicians without a cardiovascular risk factor.

Cardiovascular Risk Factor	Baseline	Follow up
	*Cholesterol unawareness*	
	*Odds ratio and 95% confidence interval*	
**Age ≥ 65 years**	0.74 (0.69, 0.80)	1.40 (1.31, 1.51)
**Hypertension Treatment**	0.63 (0.58, 0.69)	0.90 (0.83, 0.97)
**Hyperlipidemia Treatment**,	0.34 (0.23, 0.50)	0.19 (0.16, 0.22)
**Diabetes Mellitus**	0.62 (0.52, 0.74)	1.35 (1.23, 1.49)
**BMI ≥ 25 kg/m^2^**	1.08 (1.02, 1.14)	1.08 (1.01, 1.15)
**Sedentary Lifestyle**	1.27 (1.19, 1.35)	1.63 (1.54, 1.72)
**Family History of Myocardial Infarction**	0.93 (0.86, 1.01)	0.81 (0.74, 0.88)
**Former Smoker**	1.12 (1.05, 1.18)	1.13 (1.06, 1.20)
**Current Smoker**	1.55 (1.40, 1.70)	1.71 (1.48, 1.97)

* Odds ratio based on physicians that had information on cholesterol awareness at either time point (follow up N=21,860)

**Table 4.** Odds of Being Unaware of Cholesterol at Baseline and Follow-up by Smoking History.

	Unaware of Cholesterol at Baseline and follow-up	

Smoking History	OR	95% CI
**Follow-up Questionnaire Reported Current Tobacco User**	1.71*	1.47 — 1.99
**Follow-up Questionnaire Reported Past Tobacco User**	1.16*	1.08 — 1.24
**Reported Current Tobacco User baseline and Follow-up**	1.42†	1.21 — 1.67

Abbreviations: OR = Odds Ratio; CI = Confidence Interval

* referent group physicians that never smoked

† referent group physicians that never smoked at both baseline and follow-up
